# Micellar eco-friendly HPLC method for simultaneous analysis of ternary combination of aspirin, atorvastatin and ramipril: application to content uniformity testing

**DOI:** 10.1186/s13065-023-00929-y

**Published:** 2023-03-15

**Authors:** Nora A. Abdallah, Amina M. El-Brashy, Fawzia A. Ibrahim, Mohamed I. El-Awady

**Affiliations:** 1 Department of Pharmaceutical Analytical Chemistry, Faculty of Pharmacy, Mansoura University, Mansoura, 35516 Egypt; 2 Department of Pharmaceutical Chemistry, Faculty of Pharmacy, Delta University for Science and Technology, International Coastal Road, Gamasa, Mansoura, 11152 Egypt

**Keywords:** Micellar HPLC, Aspirin, Atorvastatin, Ramipril, Laboratory prepared tablet, Content uniformity testing

## Abstract

**Background:**

Cardiovascular disease medications such as aspirin (ASP), statins like atorvastatin (ATR), and blood pressure-lowering drugs including ACE inhibitors like ramipril (RAM) have been included in the World Health Organization (WHO) Essential Medicines List (EML) for many years. Therefore, there is a strong demand to develop a simple, rapid, and sensitive analytical method that can detect and quantitate the ternary mixture of these analytes in pharmaceutical preparations in a short run time. Lately, the analytical community focused on eliminating or reducing hazardous chemicals and solvents usage.

**Results:**

A green, fast, selective, and cost-effective micellar HPLC method was established and validated for the concurrent determination of ternary combination of ASP, ATR, and RAM in the pure form and pharmaceutical preparations. Resolution of the three drugs was achieved by using a monolithic column and a micellar mobile phase consists of 0.3% triethylamine (TEA) in 90: 10 an aqueous solution of 0.12 M sodium dodecyl sulfate (SDS): n-propanol, (v/v). The pH was adjusted to 2.5 using orthophosphoric acid and a flow rate of 1.5 mL/min. was applied. To ensure method reproducibility, Valsartan (VAL) was utilized as an internal standard (IS). The UV detection of the studied drugs was performed at 210 nm. Good linearity for the three drugs was obtained over the concentration ranges of 1.0-200.0 mg/mL, 0.5-200.0 mg/mL, and 5.0-100.0 mg/mL with correlation coefficients of 0.9998,0.9999 and 0.9999 for ASP, ATR, and RAM respectively. The method sensitivity was revealed by the relatively small values of limits of detection (LOD) (0.19, 0.13 and 0.30 mg/mL) and limits of quantitation (LOQ) (0.63, 0.44 and 0.99 mg/mL) for ASP, ATR, and RAM, respectively. The retention times of ASP, ATR and RAM were 1.50, 2.3 and 4.3 min., respectively.

**Conclusions:**

The suggested technique was employed for the analysis of the three drugs in their prepared tablets maintaining the recommended pharmaceutical ratio without any interference from excipients. The method was further extended to content uniformity testing of RAM. The results were validated according to international council for harmonisation (ICH) guidelines.

## Introduction

**Fig. 1:**
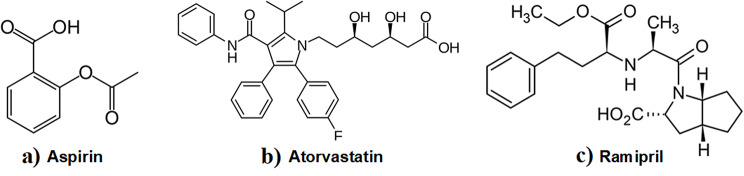
The structural formulae of the studied drugs: (a) Aspirin (b) Atorvastatin (c) Ramipril

Aspirin (ASP): (Fig. 1a) is chemically named 2-Acetyloxybenzoic acid [1]. It has been used mainly in the relief of pain, inflammation and in some fever cases. ASP is used in cardiovascular disorders due to its ability to inhibit platelet aggregation [2].

Atorvastatin (ATR); (Fig. 1b) is chemically named R-R*,R*-2-(4-Fluorophenyl)-b,d-dihydroxy-5-(1-methylethyl)-3-phenyl-4-(phenylamino)carbonyl-1 H-pyrrole-1-heptanoic acid [1]. It is used in the treatment of hyperlipidemia, as it regulates the lipid by its action on plasma lipids and reduces LDL cholesterol. Atorvastatin can be effective as adjunctive therapy in patients with homozygous familial hypercholesterolemia who have some LDL-receptor function. It was also used for primary and secondary prophylaxis of cardiovascular incidents in patients with numerous risk factors [2].

Ramipril; (Fig. 1c) is chemically (2 S,3aS,6aS)-1-(2 S)-2-(2 S)-1-Ethoxy-1-oxo-4-phenylbutan-2-yl-amino-propanoyl-3,3a,4,5,6,6a-hexahydro-2 H-cyclopenta[b]pyrrole-2-carboxylicacid [1]. Ramipril is an Angiotensin-converting enzyme (ACE) inhibitors and is used to treat hypertension, heart failure, and after myocardial infarction. It is also used to decrease the risk of cardiovascular effects in patients with certain risk factors [2].

All the three drugs were official in British [3] and United States [4] pharmacopoeia. Cardiovascular disease medications such as aspirin, statins, and blood pressure-lowering drugs including ACE inhibitors have been included in the World Health Organization (WHO) Essential Medicines List (EML) for many years. The first year of listing for aspirin was 1990, for an ACE inhibitor was 2003 and for a statin, 2007. This application seeks to add a fixed-dose combination (FDCs) of these three types of medication “a polypill” to the EML for the prevention of recurrent events in individuals with prior heart disease or stroke.

The combination used for blood pressure and cholesterol lowering and antiplatelet treatments has been recommended as potential strategy to decrease the global burden of Atherosclerotic Cardiovascular Disease given its potential for better adherence, lower costs and improved operational logistics especially in health systems in low-resource settings [5]. Due to importance of this combination the “21st Expert Committee on the Selection and use of essential medicines” recommended a fixed-dose combination of atorvastatin, ramipril and aspirin [6].

The literature of the methods reported for the simultaneous assay of that ternary combination declares that a few number of studies have been performed including HPLC [7–10], HPTLC [11] and UV spectrophotometric [12] methods, but all the reported chromatographic methods utilized mobile phases containing large quantities of expensive and hazardous organic solvents. As far as we know, no micellar HPLC methods published for the concurrent determination of this combination either in pure synthetic mixture or in tablets. According to the above details, it was desirable to develop an isocratic, simple and green HPLC method to determine these drugs in their combined formulations in short run time.

The green analytical chemistry concept aims at saving environment. It focus on replacing the required harmful chemicals used in analytical techniques with safer substitutes, using limited energy and reducing the waste, without influencing the analytical performance [13]. The consumption of organic solvents in large amounts in liquid chromatography makes it sometimes harmful technique [14]. Replacement of the common harmful mobile phases that by greener alternatives, such as water [15], ethanol or isopropanol [16]. The usage of micellar liquid chromatographic method also is a solution as it is easy to handle, time and cost saving and neither damaging the ecosystem nor the analyst. Unlike the available HPLC techniques which depend mainly on consumption of large quantities of harmful organic solvents [17] in the mobile phase. Decreasing the usage of toxic solvents and reagents is a crucial goal in terms of environment conservation and human health [18, 19]. Assessment of the proposed method was achieved through its validation according to ICH regulations. It was successfully applied to analysis of the three drugs in pharmaceutical dosage forms and content uniformity testing. In addition, the green character of the suggested method was evaluated with the three assessment tools.

## Experimental

### Equipment

- Merck Hitachi LaChrom HPLC system model L-7100 with a Rheodyne injection valve having a 20 µL loop.
- Millipore filter membranes (SIBATA) were used for filtration of the mobile phase.
- Merck solvent L-7612 degasser was used for degassing of the mobile phase.
- Consort P-901 pH-meter was used for pH measurements.

#### Materials and reagents

All used solvents were HPLC grade.

- Aspirin, 100.05 g%, Atorvastatin, 99.78 g%, and Ramipril, 100.09 g%, were provided by El Arabeya Company for pharmaceutical & Chemical Industries (ADCO), Cairo, Egypt, delta pharma, Cairo, Egypt and Sanofi-Aventis, Pakistan respectively.
- Valsartan, 100.23 g%, (IS), was provided by Memphis Co. for pharm.
- Riedel-de Häen (Seelze, Germany) was source of Triethylamine (TEA), Sodium dodecyl sulfate (SDS) and orthophosphoric acid 85%.
- Methanol, n-propanol and acetonitrile were from Sigma-Aldrich (St. Louis, MO, USA).
- Rivo^®^ 320 mg ASP/tablet, Atorstat^®^ 10 mg ATR/tablet and Tritace^®^ 1.25 mg RAM/tablet tablets, were bought from the local pharmacy in Egypt. They were manufactured under license of El Arabeya Co. for pharmaceutical and chemical Industries (ADCO), Cairo, Egypt, delta pharma, Cairo, Egypt and Sanofi-Aventis, Pakistan, respectively.

#### Chromatographic conditions

A mobile phase consisting of 90%: 10%, v/v of aqueous solution of 0.12 M SDS: n-propanol was used. To each 100 mL of the mobile phase 0.3 mL of TEA was added, finally pH was adjusted to 2.5 using orthophosphoric acid. The stationary phase was C_8_ monolithic column. Detection was performed at 210 nm using flow rate of 1.5 mL/min. at room temperature. Valsartan was selected as the internal standard of choice.

#### Standard solutions and synthetic mixtures

Methanolic stock standard solutions containing 500 µg/mL of ASP, ATR, RAM and VAL (IS) were prepared in four flasks by dissolving 50 mg of ASP, ATR, RAM and VAL separately in 100 mL methanol. Working standard solutions were obtained by subsequent dilution of the stock solutions with the mobile phase. ASP is freshly prepared every day while other solutions remain stable for 7 days when kept in the refrigerator.

Synthetic mixture solutions were obtained by mixing certain volumes of ASP, ATR and RAM stock solutions in 100 mL volumetric flasks and diluting to the volume with mobile phase keeping the pharmaceutical ratio of 10:4:1 for ASP, ATR and RAM, respectively [6].

##### Calibration graphs

Different concentrations from ASP, ATR and RAM within the range of 1-200.0, 0.5–200.0 and 5-100 µg/mL, respectively were transferred into three sets of 10-mL volumetric flasks. Volume of VAL was added to obtain a final concentration of 10 µg/mL. The flasks were completed with the mobile phase to the mark and mixed well. 20 µL of each concentration was injected (three times) under the optimum chromatographic parameters. The average peak area ratios (Drug/I.S.) were plotted against the final concentrations of each drug in µg/mL to obtain corresponding calibration curves then the corresponding regression equations were obtained.

## Assay of ASP, ATR and RAM in synthetic mixtures

Different aliquots of the stock solutions were moved to a set of 10 mL volumetric flasks achieving the ratio of 10:4:1 for ASP, ATR and RAM, respectively. The flasks were then completed with the mobile phase. The previous steps were repeated, and the percent recoveries were then calculated from the corresponding regression equations.

## Pharmaceutical application

### Analysis of the studied drugs in tablets

Ten tablets of Rivo 320 mg, Atorstat 10 mg and tritac 1.25 mg were weighed accurately and ground to fine powder individually. A weight equivalent to 12.5, 5 and 1.25 mg of Aspirin, Atorvastatin and Ramipril, respectively were dissolved in about 80 mL of methanol in a 100 mL measuring flask with 30 min. sonication. The flask was completed with the same solvent to the mark and filtered into another 100 mL volumetric flask. The final working concentrations were 125 µg/mL aspirin, 50 µg/mL of atorvastatin and 12.5 µg/mL of ramipril. Different volumes of the filtrate were analyzed with the previous mentioned procedures and the nominal contents were computed from the plotted calibration curves.

### Application of the proposed method to content uniformity testing for Tritace^®^ 1.25 mg RAM/tablet

The suggested technique was capable of testing the content uniformity of RAM in Tritace 1.25 mg RAM tablets. Content uniformity test is needed for the tablets containing ≤ 25 mg active ingredient. Owing to the small content of RAM in tablet (1.25 mg), this test can be applied for RAM uniformity testing. The test steps were followed according to the USP procedure [4] and percentage recoveries were computed by from the regression equation of RAM.

## Results and discussion

**Fig. 2:**
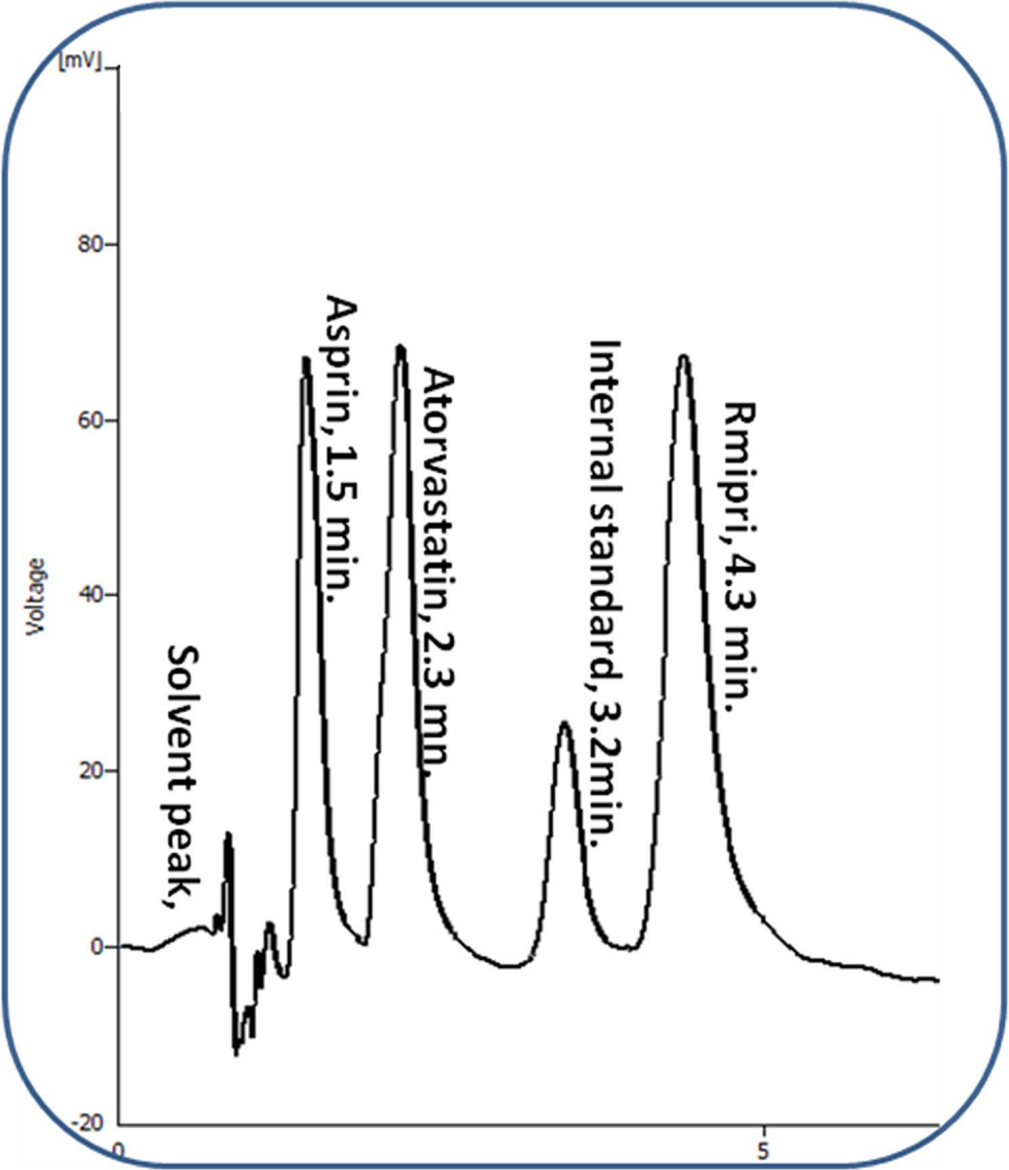
Typical chromatogram of 60.0 µg/mL ASP, 60 µg/mL ATR, 60 µg/mL RAM and 10.0 µg/mL VAL (I.S) using the optimum chromatographic conditions.

The simultaneous separation of ASP, ATR and RAM ternary mixture was carried out using miceller HPLC-UV analysis. After parameters investigation, good separation was resulted as illustrated in Fig. 2. Well-defined peaks for a synthetic mixture of the three studied drugs using the optimized micellar HPLC method. The retention times for ASP, ATR, VAL and RAM were 1.5, 2.3, 3.2 and 4.3 min., respectively, so it was found that the total runtime did not exceed 5 min. The suggested technique has superiority over other published methods. Less amount of green organic modifier was needed in the proposed method, which complies with green analysis. Shorter elution time and higher sensitivity were obtained in the current study. It is also convenient for content uniformity testing due to its ability to measure the peak area with sufficient accuracy.

## Optimization of the chromatographic parameters

Various experimental trials were made to obtain sharp symmetrical peaks as summarized in the following subsections:

### Column choice

Three columns were tried including: Shim-pack VP-ODS column C_18_ (250 mm x 4.6 mm i.d., 5 μm particle size), Onyx monolithic C_18_ 100 mm x 4.6 mm i.d., 2 μm pore size highly porous column and Onyx monolithic C_8_ 100 mm x 4.6 mm i.d., 2 μm pore size highly porous column. The study revealed that the last column showed symmetrical sharp peaks with the highest resolution, so it was the column of choice.

### Wavelength selection

**Fig. 3:**
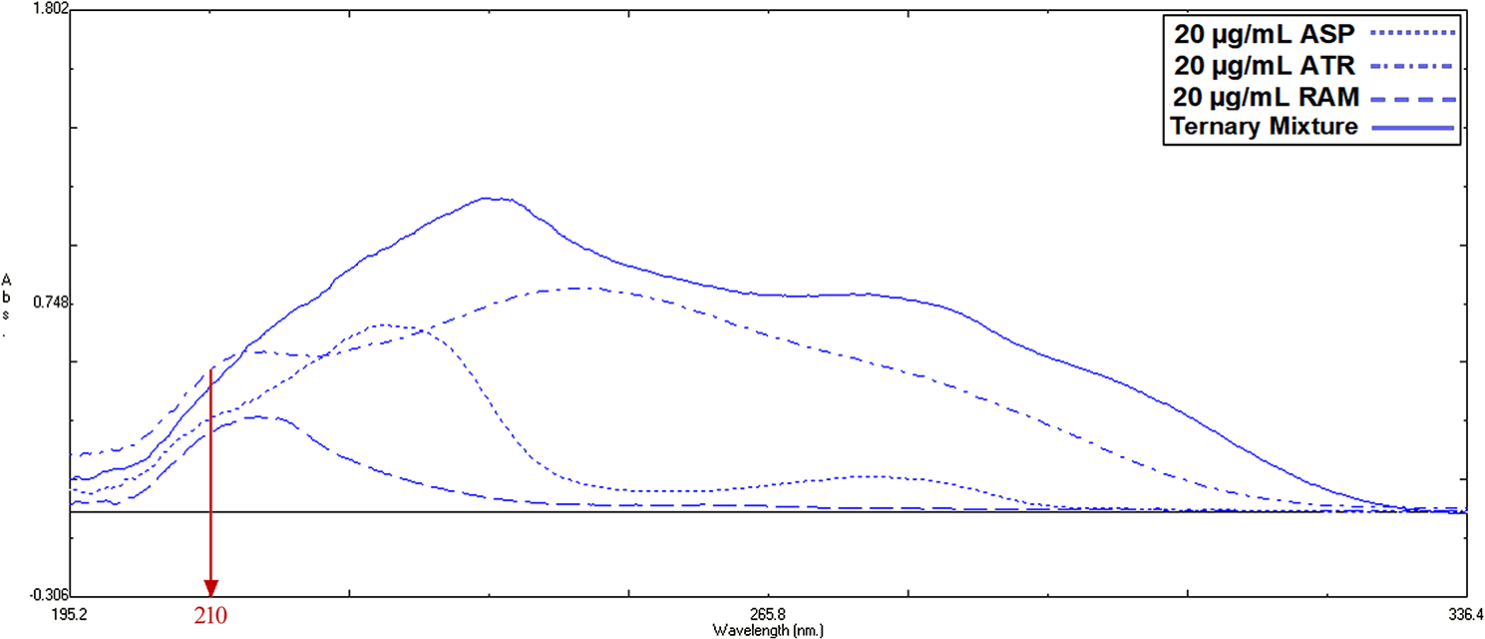
Zero-order UV spectrophotometric spectra of 20 µg/mL ASP, ATR, RAM and ternary mixture in methanol

Three wavelengths were tried 210,220 and 230; a wavelength of 210 nm was the most suitable one showing the highest sensitivity because the RAM was a weak UV-absorbing compound (Fig. 3).

### Mobile phase composition

The partition coefficient and logP were reported to be -1.1 and pk_a_ = 3.5 for ASP, for ATR logP = 6.36 and pk_a_ = 4.46 and for RAM logP = 3.32 and pk_a_ is 3.1, 5.6 [1]. Large difference between the hydrophobicities of the analytes made micellar mobile phase is appropriate for their separation. To improve the separation efficiency, TEA and short chain alcohol were added [20]. By changing the organic modifier type and concentration, SDS concentration, and pH we reached the optimum conditions as summarized in Table 1.

**Table 1:** Effect of experimental parameters on the number of theoretical plates

Parameter		Number of theoretical plates (N)			Resolution, *R*_S_		Selectivity, *α*	
		ASP	ATR	RAM	ASP/ATR	ATR/RAM	ASP/ATR	ATR/RAM
% Concentration n- propanol (v/v)								
	6	770	1271	746	2.9	4.2	3.5	2.78
	8	510	931	1005	2.7	4.7	3.7	2.9
	10	504	971	1317	2.7	4.6	3.1	2.7
	12	465	892	1336	1.9	4.42	3.6	3.4
	14	423	743	1402	3.5	3.6	16	2.7
	15	567	1112	363	5.1	2.7	18	2.2
**Concentration of SDS, M**								
	0.05	467	1045	395	4.6	8	3	3.2
	0.08	970	1766	1172	4.36	7.2	3.4	3.2
	0.1	659	1229	1491	3	5	3.3	3.3
	0.12	515	869	1485	2	4.44	4	3.5
	0.15	480	859	1158	1.7	3.6	3.75	3.3
	0.18	414	658	1143	1.5	2.2	6	3.5
**pH of the medium**								
	2.5	515	971	1485	3.2	5	3	2.66
	3	651	606	1173	2.2	5.87	3.75	4.1
	4	308	1207	207	0.32	6.35	1.17	26.76
	5	Overlapped		343				
	6	Overlapped peaks						
**Flow rate, mL/min.**								
	0.8	417	1094	forked	2.6	3.36	4.42	2.64
	1	469	987	1380	2.38	4.4	4.16	3.12
	1.2	458	974	1376	2.8	4.16	4.24	3.76
	1.5	515	871	1485	2.54	4.2	4	3.37
	2	473	964	1210	2.26	4.34	5.5	3.36

### Type of organic modifier

The effect of different organic modifiers, including methanol, n-butanol and n-propanol, was studied in order to achieve the best separation of the studied drugs. N-propanol gave well-resolved sharp peaks within a reasonable short analysis time (less than 4.5 min.) so it was the organic modifier of choice.

### Organic modifier concentration

Eluents containing 6 to 15% v/v of n-propanol were tried. The optimum chromatographic performance was attained using 10% v/v of n-propanol as it yields the maximum NTP with good *R*s. Lower concentrations result in broadening of RAM peak, so decreasing the sensitivity. While higher concentrations lead to overlapping of ASP peak with solvent one. Regarding resolution and number of theoretical plates of the drugs, the optimum n-propanol concentration was selected to be 10% v/v.

### SDS concentration

Different concentrations of SDS were tried using mobile phases containing SDS in the range 0.05 to 0.18 M, while keeping the concentration of n-propanol and pH constant. The retention times of RAM increase and sensitivity decrease significantly upon decreasing the concentration of SDS. This indicated that the best chromatographic performance was using 0.12 M SDS regarding resolution and number of theoretical plates of the studied drugs. Concentrations less than 0.08 M SDS result in significant increasing in the retention time and decreasing in sensitivity of RAM, while concentrations higher than 0.12 M SDS result in decreasing the resolution of the peaks.

### pH

The effect of varying mobile phase pH on the chromatographic performance of the developed method was studied using different mobile phases with pH ranging from of 2 to 6 (using H_3_PO_4_ or TEA), while keeping the concentration of n-propanol and SDS constant. It was found that the sensitivity and the resolution of three drugs decrease upon decreasing the pH below 2.5. At pH above 3, significant decrease in the sensitivity and increase in retention time of both ATR and RAM. pH 2.5 was found to be the most appropriate pH.

### Flow rate

The flow rate was changed from 0.8 to 2 mL/min. and a flow rate 1.5 ml/min. was found to be optimum for good separation in a reasonable time.

### The nature of internal standard

Both the internal standard and drug are analyzed simultaneously to compensate for any possible variation during the entire process of sample preparation and analysis. Different internal standards including valsartan, clopidogrel, rivaroxaban and amlodipine were investigated. Valsartan (VAL) was the internal standard of choice showing the highest resolution from the peaks of the three drugs.

The final optimized conditions were as follow: an aqueous solution containing 0.12 M SDS, 10% (v/v) n-propanol and 0.3% (v/v) TEA, adjusted to pH 2.5 using orthophosphoric acid. The mobile phase was pumped at a flow rate of 1.5 mL/min. and UV detector was set at 210 nm.

#### Method validation

Validation of the proposed method was accomplished as per ICH recommendations [21] as follows:

### Linearity, range, limit of quantitation (LOQ) and limit of detection (LOD)

Linearity was studied for ASP, ATR and RAM by plotting relationship between peak area ratio [drug/I.S.] and each drug concentrations (C) in µg/mL. Table 2 presents the equations of the regression lines, linearity ranges and the analytical data of the calibration curves. Statistical analysis [22] of the data are abridged in Table 2.

**Table 2:** Analytical performance data for the determination of the ASP, ATR and RAM by the proposed Micellar HPLC method

Parameter	ASP	ATR	RAM
Range (µg/mL)	0.1–200	0.5–200	5.0-100
Intercept (a)	0.0077	0.0557	-0.1199
Slope (b)	0.0337	0.0498	0.0734
Correlation coefficient (r)	0.9998	0.9999	0.9999
S.D. of residuals (S_y/x_)	0.0027	0.00335	0.00148
S.D. of intercept (S_a_)	0.00005	0.00005	0.00007
S.D. of slope (S_b_)	0.00001	0.00002	0.00002
Regression equation	*P = 0.0337 C + 0.0077	*P = 0.0498 C + 0.0557	*P = 0.0734 C − 0.1199
Percentage relative standard deviation, % RSD	1.315	1.027	1.078
Percentage relative error, % Error	0.412	0.309	0.407
Limit of detection, LOD (µg/mL)	0.19	0.13	0.30
Limit of quantitation, LOQ (µg/mL)	0.63	0.44	0.99

*P is the peak area ratio, C is the concentration of the drug in µg/mL and r is the correlation coefficient

The limit of quantitation (LOQ) and the limit of detection (LOD) were calculated practically according to ICH recommendations [21]. Limit of detection (LOD) and limit of quantification (LOQ) results are given in Table 2.

### Accuracy

The accuracy of the proposed method was evaluated through comparing the results of assay of the proposed method with those obtained using the comparison method [9]. The comparison method was a RP-HPLC using C-18 column, a mobile phase composed of methanol and acetate buffer in the ratio (70:30 v/v) at pH to 3.1.

When comparing the proposed approach with a comparison method, student’s *t*-test and variance ratio *F*-test [22] revealed no-significant difference as shown in Table 3.

**Table 3:** Assay results for determination of ASP, ATR and RAM drugs in pure forms by the proposed micellar HPLC method

Compound	Proposed method		Comparison method [9]
	Amount taken, (µg/mL)	% Found	% Found
ASP	1	100.20	99.37
	40	100.59	100.83
	100	99.94	99.69
	150	101.91	
	200	98.85	
Mean ± SD		100.31 ± 1.11	99.69 ± 0.77
*t-*test		0.45 (2.45)	
*F*- test		2.09 (19.25)	
ATR	0.5	99.00	99.05
	40	100.63	101.34
	100	99.74	99.49
	150	101.05	
	200	99.55	
Mean ± SD		99.99 ± 0.83	99.96 ± 1.22
*t*-test		0.05 (2.45)	
*F*-test		2.13 (6.95)	
RAM	5	100.86	101.03
	20	99.53	98.73
	60	99.43	100.46
	80	98.94	
	100	100.72	
Mean ± SD		99.91 ± 0.85	100.07 ± 1.21
*t*-test		0.25 (2.45)	
*F*-test		2.00 (6.95)	

### Precision

Repeatability and intermediate precision of the proposed method were tested. The repeatability was examined by testing three different concentration of each drug three replicates in the same day and in three consecutive days for intermediate precision. The studied concentrations were: 80.0,100.0,120.0 µg/mL for ASP & ATR and 40.0,050.0,and 60.0 µg/mL for RAM. The results indicate acceptable repeatability and intermediate precision of the proposed method as shown in Table 4.

**Table 4:** Precision data for the determination of ASP, ATR and RAM by the proposed Micellar HPLC method

Am. taken, µg/mL		ASP concentration			ATR concentration			RAM concentration		
		80.0	100.0	120.0	80.0	100.0	120.0	40.0	50.0	60.0
Intraday	% Found	99.14	99.26	99.45	100.46	100.45	99.85	100.46	99.75	98.85
		99.62	98.83	99.12	100.73	99.75	100.37	101.32	99.93	98.77
		99.35	98.46	98.20	101.52	99.24	100.48	101.22	99.57	99.18
	Mean	99.37	98.85	98.92	100.90	99.81	100.23	101	99.75	98.93
	±SD	0.24	0.40	0.65	0.55	0.61	0.34	0.47	0.18	0.22
	%RSD	0.24	0.40	0.66	0.55	0.61	0.34	0.47	0.18	0.22
	%Error	0.14	0.23	0.38	0.32	0.35	0.19	0.27	0.10	0.13
Interday	% Found	100.13	99.36	99.82	99.66	100.78	100.46	99.68	100.58	99.87
		99.78	99.03	98.96	100.04	101.26	99.82	101.24	101.17	101.00
		99.94	99.58	99.91	100.11	101.04	101.03	100.52	100.34	100.03
	Mean	99.95	99.32	99.56	99.94	101.03	100.44	100.48	100.70	100.30
	±SD	0.18	0.28	0.52	0.24	0.24	0.61	0.78	0.43	0.61
	%RSD	0.18	0.28	0.53	0.24	0.24	0.60	0.78	0.42	0.61
	%Error	0.10	0.16	0.30	0.14	0.14	0.35	0.45	0.25	0.35

### Robustness

The robustness was evaluated, and some parameters showed an acceptable range for variation including n-propanol concentration (10.0 ± 0.5% v/v), SDS concentration (0.12 M ± 0.005) and pH of the mobile phase (2.5 ± 0.2). As shown in Table 5, these slight changes did not affect the peak area ratios or the retention times of the three analytes.

**Table 5:** Robustness of the proposed method using ASP (100.0 µ/mL), ATR (100.0) and RAM (50.0 µ/mL)

Parameter	% Found, %		
	ASP	ATR	RAM
pH			
2.3	98.40	101.14	99.96
2.5	98.22	100.11	99.64
2.7	99.12	99.15	100.14
Mean	98.58	100.13	99.91
± S.D.	0.48	0.99	0.25
% RSD	0.48	0.99	0.25
% Error	0.28	0.57	0.15
**Concentration of n-Propanol (%v/v)**			
9.5	99.48	100.23	101.76
10	99.45	99.67	100.44
10.5	100.34	100.78	101.06
Mean	99.76	100.23	101.09
± S.D.	0.51	0.56	0.66
% RSD	0.51	0.55	0.65
% Error	0.29	0.32	0.38
**SDS concentration, M**			
0.115	99.75	100.45	99.47
0.12	98.65	99.67	100.34
0.125	99.88	99.96	100.12
Mean	99.43	100.03	99.98
± S.D.	0.68	0.39	0.45
% RSD	0.68	0.39	0.45
% Error	0.39	0.23	0.26

### Selectivity and specificity

The suggested technique was able to determine ASP, ATR or RAM in presence of each other with high accuracy without any interference.

The proposed method specificity was tested by observing any interference resulted from the used dosage forms excipients such as: microcrystalline cellulose, magnesium stearate, citric acid anhydrous, colloidal silicon dioxide, butylated hydroxyanisol, talc, croscarmellose sodium, lactose monohydrate, maize starch, hypromellose, pregelatinised, sodium stearyl fumarate. All these compounds did not show any interference with the results of the suggested technique as shown in Table 6.

**Table 6:** Assay results for the determination of the ASP, ATR and RAM in prepared tablet

Compound	Proposed method		Comparison method [9]
	Amount taken, (µg/mL)	% Found, %	% Found, %
ASP	62.5	98.95	100.52
	125	101.02	99.45
	187.5	99.77	100.18
Mean ± SD		99.91 ± 1.31	100.05 ± 0.547
*t*-test		0.20 (2.78)	
*F*- test		3.64 (19.00)	
ATR	25	99.16	98.54
	50	99.91	101.18
	75	99.67	99.63
Mean ± SD		99.58 ± 0.383	99.78 ± 1.33
*t*-test		0.26 (2.68)	
*F*-test		11.99 (19.00)	
RAM	6.25	99.44	100.67
	12.5	101.22	99.42
	18.75	100.11	100.18
Mean ± SD		100.26 ± 0.90	100.09 ± 0.63
*t*-test		0.26 (2.78)	
*F*-test		2.04 (19.00)	

### System suitability

The resolution and reproducibility of the produced peaks was checked to make sure if the selected chromatographic conditions are appropriate for the analysis or not. By comparing to the reference official values, acceptable parameters were resulted as shown in Table 7.

**Table 7:** Parameters of system suitability for the proposed HPLC method for the determination of VAL and AML

Parameter	ASP/ATR	ATR/IS	IS/RAM	Reference value
**Retention time, min**	1.5	2.3	4.3	
**Capacity factor, K’**	0.36	1.09	2.9	0–10
**Symmetry factor**	0.718	0.829	1.05	~ 1
**Resolution**, ***R***_***S***_	2.67	2.4	2. 2	RS > 2
**Selectivity**, ***α***	3.0	1.75	1.52	α > 1
**Theoretical plates**, ***N***	400	690.93	603.75	

### Greenness assessment

Green analytical chemistry plays a major role in preserving and protecting the environment. The analytical Eco-Scale [23] is a green metric that is used to assess the greenness of an analytical method. It has unique advantages compared to other metrics. It is easy to calculate its score that makes comparison of analytical procedures very easy [24]. It includes varied aspects of environmental impact in its assessment procedure. It is calculated by allotting penalty points to any factor in the analytical procedure. The analytical Eco-Scale score is calculated by subtracting penalty points from the basis of 100 points. A score of 100 represents an ideal green analysis. The higher the value is, the greener the method will be [25–27].

The comparison between the analytical Eco-Scale values of the proposed approach and reported methods [7–9] is carried out and results are shown in Table 8. It was found that the proposed method is greener than the reported methods giving a score of 72.

**Table 8:** The penalty points of the proposed methods according to the analytical Eco-Scale

Reagents/ Instruments				Penalty points			
Reagent	Number of pictograms	Word sign	Reagent, penalty points	Proposed method	Reported method [7]	Reported method [8]	Reported method [9]
Methanol	3	Danger	6		6 × 1 = 6	6 × 2 = 12	6 × 2 = 12
Acetonitrile	2	Danger	4		4 × 2 = 8	4 × 2 = 8	
n- propanol	2	Danger	4	2 × 4 = 8			
Glacial acetic acid	2	Danger	4				2 × 4 = 8
TEA	3		3	3 × 1 = 3			
o-phosphoric acid	2		2	2 × 1 = 2	2 × 2 = 4	2 × 2 = 4	2 × 1 = 2
Sodium acetate	1	Irritant	2				2 × 2 = 4
HPLC							
Occupational hazard, Analytical process hermitization				0	0	0	0
Waste Flow rate x run time			1–10 = 3 > 10 = 5	3	5	5	5
Total penalty points				16	23	29	31
Analytical Eco- Scale total score				84	77	71	69

**Fig. 4:**
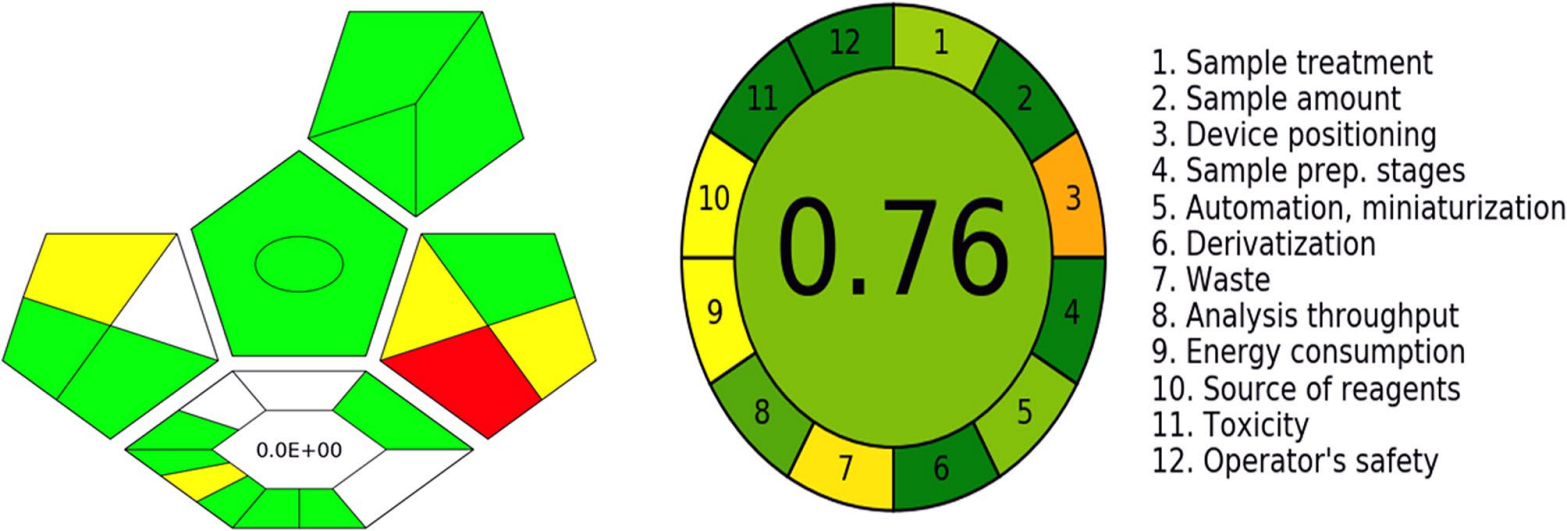
Complex GAPI pentagram and AGREE result of the proposed method.

The method greenness was further assessed using complex GAPI [28] and AGREE [29] tools. Figure 4 showed GAPI pentagram and AGREE result. The AGREE score was found to be 0.76 indicating the greenness of the proposed method.

#### Applications

### Analysis of studied drugs in synthetic mixtures

The developed technique was successfully used to the concurrent determination of ASP, ATR and RAM in laboratory-prepared mixtures regarding their recommended pharmaceutical ratio, 10:4:1 respectively (Fig. 2). The statistical assessment of the results confirms no significant difference with those of the comparison method [9]. Good recoveries were obtained and abridged in Table 9.

**Table 9:** Assay results for the determination of the ASP, ATR and RAM drugs in synthetic mixtures in ratios of 10:4:1 (w/w) by the proposed HPLC method

Compound	Proposed method		Comparison method [9]
	Amount taken, (µg/mL)	% Found, %	% Found, %
ASP	100	99.14	101.02
	140	101.10	100.45
	200	99.71	100.58
Mean ± SD		99.98 ± 1.01	100.35 ± 0.73
*t-*test		1.15 (2.78)	
*F*- test		11.39 (19.00)	
ATR	40	99.33	99.67
	56	100.84	100.28
	80	99.97	99.78
Mean ± SD		100.05 ± 0.76	99.91 ± 0.33
*t-*test		0.29 (2.78)	
*F*-test		5.43 (19.00)	
RAM	10	101.57	100.03
	14	98.59	98.56
	20	100.31	101.46
Mean ± SD		100.16 ± 1.50	100.017 ± 1.45
*t-*test		0.12 (2.78)	
*F*-test		1.06 (19.00)	

### Dosage forms

**Fig. 5:**
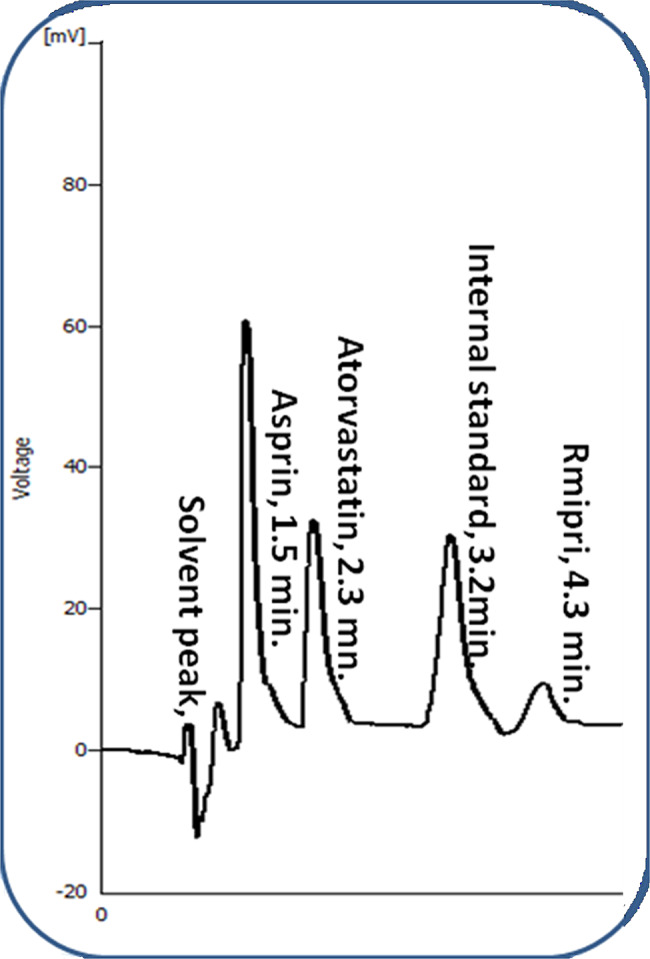
Typical chromatogram of 187.5 µg/mL ASP, 75 µg/mL ATR And 18.75 µg/mL RAM in the prepared tablet using 10.0 µg/mL VAL (I.S) using the optimum chromatographic conditions

The three drugs were investigated in their laboratory prepared tablets using the proposed method as shown in Fig. 5. The results of the assay agreed with those obtained with the comparison method [9] as shown in Table 6.

### Content uniformity

The suggested technique was ideally suitable for content uniformity testing of RAM. Table 10 showed that acceptance value (AV) was smaller than the maximum allowed acceptance value (L1). The results indicated excellent drug uniformity.

**Table 10:** Results of content uniformity testing of RAM in Ttritace® tablets using the applied method

Parameter	Tablet no.	Percentage of the label claim
Data	1	102.49 g%
	2	101.21 g%
	3	102.11 g%
	4	98.52 g%
	5	100.44 g%
	6	98.59 g%
	7	102.54 g%
	8	98.18 g%
	9	102.80 g%
	10	99.85 g%
**Mean**		100.67 g%
**S.D.**		1.81
**% RSD**		1.80
**% Error**		0.569
**Acceptance value (AV)**[4]	4.35	
**Max. allowed AV (L1)**[4]	15.0	

Tritace® tablets: Labeled to contain 1.25 mg ramipril per tablet; manufactured by Sanofi Aventis.

## Conclusion

New, green, accurate and rapid micellar HPLC method was designed for the concurrent determination of ASP, ATR and RAM in their ternary mixtures. The correlation coefficients are 0.9998, 0.9999 and 0.9999 for ASP, ATR and RAM respectively. The recommended method had practical limits of detection of 0.19, 0.13 and 0.30 µg/mL and limits of quantitation of 0.63, 0.44 and 0.99 µg/mL, respectively. It could be applied to the assay of the three drugs in their combined tablets. It was also very suitable to be used in content uniformity testing for RAM. The suggested approach could be considered a suitable alternative for the previous harmful methods.

## Data Availability

Most of the data of this study are included within the article. Other supplementary data are available from the corresponding author upon request.

## Abbreviations

ASP: Aspirin
ATR: Atorvastatin
RAM: Ramipril
TEA: Triethylamine
SDS: Sodium dodecyl sulfate
VAL: Valsartan
IS: Internal standard
LOD: Limits of detection
LOQ: Limits of quantitation
ICH: International council for harmonisation
ACE: Angiotensin-converting enzyme
WHO: World health organization
EML: Essential medicines list
FDC: Fixed-dose combination.
